# Benefits of a transfer clinic in adolescent and young adult kidney transplant patients

**DOI:** 10.1186/s40697-015-0081-6

**Published:** 2015-12-15

**Authors:** Rory F. McQuillan, Alene Toulany, Miriam Kaufman, Jeffrey R. Schiff

**Affiliations:** Division of Nephrology and Department of Medicine, University Health Network, Toronto, Ontario Canada; Division of Adolescent Medicine, Department of Paediatrics, Hospital for Sick Children, University of Toronto, Toronto, Ontario Canada; Multi-Organ Transplant Program, University Health Network, Toronto, Ontario Canada; The Transplant and Regenerative Medicine Centre, Hospital for Sick Children, University of Toronto, Toronto, Ontario Canada; Good 2 Go Transition Program, Hospital for Sick Children, Toronto, Ontario Canada; Toronto General Hospital, 200 Elizabeth Street, 8N-819, Toronto, Ontario M5G 2C4 Canada; Hospital for Sick Children, 525 University Avenue, Toronto, Ontario M5G 1X8 Canada; Toronto General Hospital, 585 University Avenue, 11 PMB 185, Toronto, Ontario M5G 2N2 Canada

**Keywords:** Transplant, Transition, Transfer, Health care, Adherence

## Abstract

**Background:**

Adolescent and young adult kidney transplant recipients have worse graft outcomes than older and younger age groups. Difficulties in the process of transition, defined as the purposeful, planned movement of adolescents with chronic health conditions from child to adult-centered health care systems, may contribute to this. Improving the process of transition may improve adherence post-transfer to adult care services.

**Objective:**

The purpose of this study is to investigate whether a kidney transplant transfer clinic for adolescent and young adult kidney transplant recipients transitioning from pediatric to adult care improves adherence post-transfer.

**Methods:**

We developed a joint kidney transplant transfer clinic between a pediatric kidney transplant program, adult kidney transplant program, and adolescent medicine at two academic health centers. The transfer clinic facilitated communication between the adult and pediatric transplant teams, a face-to-face meeting of the patient with the adult team, and a meeting with the adolescent medicine physician. We compared the outcomes of 16 kidney transplant recipients transferred before the clinic was established with 16 patients who attended the clinic. The primary outcome was a composite measure of non-adherence. Non-adherence was defined as either self-reported medication non-adherence or displaying two of the following three characteristics: non-attendance at clinic, non-attendance for blood work appointments, or undetectable calcineurin inhibitor levels within 1 year post-transfer.

**Results:**

The two groups were similar at baseline, with non-adherence identified in 43.75 % of patients. Non-adherent behavior in the year post-transfer, which included missing clinic visits, missing regular blood tests, and undetectable calcineurin inhibitor levels, was significantly lower in the cohort which attended the transfer clinic (18.8 versus 62.5 %, *p* = 0.03). The median change in estimated glomerular filtration rate (eGFR) in the year following transfer was smaller in the group that attended the transition clinic (−0.9 ± 13.2 ml/min/1.73 m^2^) compared to those who did not (−12.29 ± 14.9 ml/min/1.73 m^2^), *p* = 0.045.

**Conclusions:**

Attendance at a single kidney transplant transfer clinic was associated with improved adherence and renal function in the year following transfer to adult care. If these changes are sustained, they may improve long-term graft outcomes for adolescent kidney transplant recipients.

## Résumé

**Contexte:**

L’évolution favorable du greffon est plus souvent compromise chez les adolescents et les jeunes adultes transplantés du rein que chez les enfants et les adultes ayant subi la même intervention. Ces jeunes patients qui sont en général atteints de maladies chroniques, rencontrent des difficultés au cours de la période de transition entre le moment de leur transfert des unités de soins pédiatriques vers les unités de soins pour adultes, et celles-ci pourraient contribuer à ce pronostic défavorable. Des améliorations apportées au processus de transition pourraient favoriser l’adhésion de ces jeunes patients à leur protocole de traitement à la suite leur transfert dans les services de soins pour adultes.

**Objectif:**

Le but de cette étude est de vérifier si la fréquentation d’une clinique de transfert pouvait améliorer l’adhésion des adolescents et des jeunes adultes greffés du rein à leur traitement, après leur transfert d’un établissement pédiatrique vers des services de soins pour adultes.

**Méthodes:**

Nous avons développé, au sein de deux centres universitaires de santé, deux cliniques conjointes de transfert pour les transplantés du rein. Ces cliniques étaient formées d’un programme de transplantation rénale pédiatrique, d’un programme de greffe rénale pour adultes et d’une clinique de médecine adolescente. La mise en place d’une clinique de transfert a facilité la communication entre les équipes de transplantation pour adultes et pédiatriques, a permis aux patients adolescents de rencontrer les équipes de transplantation pour adultes et de rencontrer des spécialistes de la médecine adolescente. Nous avons comparé les résultats de 16 jeunes greffés du rein qui avaient été transférés dans les centres de soins pour adultes avant la mise en place de la clinique de transfert avec les résultats de 16 patients qui ont fréquenté la clinique de transfert avant leur transition vers les unités de soins pour adultes. Le principal résultat a été une mesure composite d’adhésion au traitement. La non-adhésion a été définie soit par l’aveu de la part du patient de sa non-observance du traitement médicamenteux, soit par la manifestation de deux des trois comportements suivants dans le suivi du patient : la non-fréquentation de la clinique de transfert, le défaut de se présenter aux rendez-vous pour les analyses sanguines ou un niveau indécelable des inhibiteurs de calcineurine dans l’année suivant le transfert vers les services de soins pour adultes.

**Résultats:**

Les patients des deux groupes présentaient des caractéristiques similaires au début de l’étude, et 43,75 % d’entre eux avaient admis ne pas adhérer entièrement au traitement. Le nombre de comportements identifiés comme signes de non-adhésion au traitement tels que manquer des rendez-vous à la clinique de transfert, ne pas se présenter pour les analyses sanguines ou un niveau d’inhibiteurs de la calcineurine indécelable dans l’année suivant le transfert, étaient nettement inférieurs dans la cohorte de patients qui fréquentait la clinique de transfert que dans celle des patients qui avaient été transférés directement dans les services de soins pour adultes (18,8 % versus 62,5 %, *p* = 0,03). Qui plus est, les patients ayant fréquenté la clinique de transfert présentaient une variation médiane plus faible du débit de filtration glomérulaire (−0,9 ± 13,2 ml/min/1,73 m^2^) lorsque comparée à celle du groupe ayant été transféré directement (−12,2 ± 14,9 ml/min/1,73 m^2^), *p* = 0,045.

**Conclusions:**

Le fait de fréquenter une clinique de transfert pour les greffés du rein, dans l’année suivant leur transfert dans un centre de soins pour adultes, donne lieu à la fidélisation des jeunes transplantés du rein à l’égard de leur traitement et ceci favorise le rétablissement de leur fonction rénale. Le maintien de ces changements de comportement pourrait améliorer le pronostic à long terme quant à l’évolution du greffon chez les adolescents et les jeunes adultes greffés du rein.

## Background

Adherence may be defined as the extent to which patients are able to follow recommendations for prescribed treatment [1, 2]. Although there is no universal method to assess adherence, information from multiple sources including the adolescent, family, health care providers, and direct measurement of medication or metabolite blood levels is most reliable [3–5]. Non-adherence is a complex and multi-factorial phenomenon that may occur at any stage during treatment. Low adherence may increase morbidity and medical complications, contributing to poorer quality of life and an overuse of the health care system [3, 6]. Examples of non-adherence may include failing to collect a prescription from a pharmacy, not taking a prescribed medication as directed, taking too much medication or skipping doses, or taking the medication at the wrong time. Adolescent and young adult patients may be non-adherent due to issues related to the complexity of a medication regimen, poor communication with a health care provider, or a number of patient-related factors such as their developmental stage, emotional issues, and family dysfunction [3]. Non-adherence with treatment after kidney transplantation is associated with poor clinical outcomes and increased health care costs [7–10]. Non-adherence may refer to multiple elements, including missing medications, taking medications incorrectly, missing clinic visits, or missing scheduled blood tests.

One quarter of all kidney transplant recipients are estimated to be non-adherent [11]. Adolescents are at particular risk of failing to fully adhere to their medication regimen [12, 13]. This may explain why their graft survival is worse than in any age group up to age 70 [14].

There are many unique developmental tasks during adolescence that may contribute to the problem of non-adherence in this group of patients. Immunosuppressive medications may induce changes in one’s body at a time when the adolescent is adjusting to one’s physique and dealing with issues of self-esteem [9]. Normal adolescent tendencies of testing independence and questioning authority may predispose them to reject medical advice and treatment. Other factors such as impulsivity and risk taking, sense of indestructibility, denial of severity of illness, and wanting to “be normal” may also contribute. In addition, developing a complex, chronic illness in childhood or adolescence negatively impacts adolescents’ development psychologically, physiologically, and socially, thereby interrupting the normative adolescent developmental processes. Therefore, the period of transition from pediatric to adult care may be of particularly high risk. For example, in a cohort of 20 patients transferred from pediatric care without a transition plan in place, Watson demonstrated a 35 % incidence of graft loss within the first 3 years [15].

Transition is defined as the purposeful, planned movement of adolescents with chronic physical and medical conditions from child-centered to adult-oriented health care systems [16, 17]. Transitioning adolescents with complex, chronic health care needs is challenging for patients, families, and health care workers [18–24]. The transition process is often inadequately planned, interrupted, and poorly coordinated. These challenges contribute to an increased risk of patient disengagement, health care dropout, and poor treatment adherence. This can lead to more emergency room visits, hospitalizations, and poor health outcomes [15, 25–27].

Several international and national policy and position statements call for transition planning and evaluation of transition outcomes [17, 24]. It has been suggested that transition programs for adolescents with chronic illness would provide key opportunities to modify non-adherent behavior and self-efficacy and improve overall health outcomes [24, 28–30]. There are virtually no studies linking improved outcomes with a specific transition intervention. In recognition of these issues, we developed a joint pediatric-adult kidney transplant transfer clinic in order to improve the transition of patients between pediatric and adult kidney transplant programs.

## Methods

### Setting

The renal transplant transfer clinic was implemented as a joint initiative between the Transplant and Regenerative Medicine Centre (TRMC) at the Hospital for Sick Children (SickKids) and the Multi-Organ Transplant (MOT) Program at University Health Network (UHN). SickKids is the largest pediatric transplant center in Canada, performing 20–25 kidney transplants per year. The MOT Program at UHN provides a broad spectrum of services currently encompassing heart, lung, liver, kidney, pancreas, and small bowel transplantation. Approximately 500 transplants are performed annually, including 150–180 kidney transplants. Follow-up care is provided to almost 5000 transplant recipients. In Ontario, government regulations mandate the transfer of care from pediatric to adult health care settings at the age of 18, regardless of time post-transplant.

Usual transition care in the pediatric kidney transplant clinic begins several years prior to transfer. Elements include most adolescent patients seeing a health care provider for part of the appointment without their parents; encouragement to learn the names and doses of their medications; coaching to be able to communicate information about their diagnosis and transplant history; and general adolescent health care that includes reproductive and contraceptive counseling, career guidance, and drug and alcohol counseling. Adolescent medicine interventions include managing comorbid mental health issues appropriately, customizing the treatment regimen when possible, providing information, ensuring family and peer support, and empowering the adolescent to overcome adherence issues though motivational interviewing and other techniques.

Prior to the establishment of the transfer clinic, patients were distributed between all adult transplant nephrologists and coordinators at UHN. Adult providers did not meet the patients prior to transfer, and the schedule for clinic visits and labs was the same as an adult patient at a comparable time post-transplant; for example, a patient 5 years post-transplant would have had routine lab tests every 3 months, with clinic visits only once or twice per year.

The kidney transplant transfer clinic was set up in 2009 by an inter-disciplinary team of pediatric and adult care providers. The goal of the transfer clinic is to enhance patient experience at the time of transition through improved care coordination and integration. The clinic provides a structured meeting place between the patient and the new adult care team in the pediatric hospital. It also allows the pediatric and adult care teams a chance to communicate face-to-face about each patient. Patients’ graduations from the pediatric clinic were also celebrated.

The transfer clinic is held twice a year in the pediatric kidney transplant clinic area. Kidney transplant recipients who will be turning 18 in the next 6 months attend the clinic. A typical clinic includes between six and nine patients, often accompanied by one or both parents. During each clinic, there is a formal discussion of each patient by the pediatric and adult kidney transplant teams. This includes a review of the patient’s history prior to transplant, post-transplant course, immunologic status, and other medical issues. Any follow-up that the patient will require with other specialists is noted, as are any issues regarding the patient’s social situation. Following this case review, the adult team is then introduced to and meets with the patient and any family members present, discusses the date of transfer and the process of the first clinic visit at UHN, and confirms contact information. They do not participate in the patient’s clinical care at that visit.

Patients and parents separately participate in small group discussions facilitated by members of the SickKids Good 2 Go Transition Program. Topics for discussion include differences in the pediatric and adult health systems; education and career plans; financial issues such as insurance, student loans and grants, and the importance of filing income tax forms; reproductive issues; and self-management and adherence. Patients also complete a MyHealth Passport and receive a “Getting Ready for Adult Care” booklet and a graduation certificate. MyHealth Passport (www.sickkids.ca/myhealthpassport) is a free online program that helps young persons create a wallet-sized card with important health information (that they can also email to themselves or others). MyHealth Passport was originally designed to improve adolescent patients’ knowledge of their health history, to give them a sense of ownership of this information, and to ensure that important information is communicated in a new or emergency situation. Three copies of the MyHealth Passport are printed for each patient; two are cut out and laminated, and they are encouraged to give the other to their student health center if they are going on to post-secondary education.

All transferred patients are now directed to a single adult transplant nephrologist and coordinator, who attend the transfer clinic. Regardless of time post-transplant, transferred patients are initially seen at least every 3 months after transfer and are asked to have routine labs drawn monthly. Depending on the time post-transplant and the patient’s clinical status, this may be more frequent than their follow-up at SickKids prior to transfer. Patients receive routine reminders of upcoming clinic visits and missed appointments are rebooked (similar to other patients), but they are not given specific reminders about blood tests. This practice was in place both before and after the initiation of the transfer clinic.

### Study design

This was a retrospective cohort study, approved by the research ethics board of UHN and SickKids, to examine the effect of the transfer clinic on patient adherence. The clinical and research activities being reported are consistent with the Principles of the Declaration of Istanbul as outlined in the Declaration of Istanbul on Organ Trafficking and Transplant Tourism.

Patients were divided by era; those who transferred prior to 2009 did so before the transfer clinic existed; those who transferred after 2009 attended the transfer clinic. Transition preparation was otherwise the same. The study population therefore consisted of 32 consecutive patients who had transferred to adult care in the Greater Toronto Area at 18 years of age between July 2007 and June 2011. The study hypothesis was that adherence of patients who had attended the clinic would be better than those who did not.

Baseline information recorded at the time of transfer included age, gender, age at end-stage renal disease (ESRD) diagnosis, age at transplant, histocompatibility data, type of transplant (either deceased or living donor), number of transplants, documented self-reported non-adherence in pediatric care (defined as missed medication doses), rejection episodes, and serum creatinine.

Non-adherent behavior was a composite measure defined as either self-reported medication non-adherence or displaying two of the following three characteristics: any non-attendance at clinic, non-attendance for blood work appointments, or undetectable calcineurin inhibitor levels. This was captured electronically in the case of missed appointments, non-attendance at clinic, and undetectable calcineurin inhibitor levels. All patients are asked about medication non-adherence at their clinic visits. This was documented electronically via the Organ Transplant Tracking Record (OTTR™, OTTR Chronic Care Solutions, Omaha, NE, USA).

The primary outcome was the difference in the number of patients exhibiting non-adherent behavior in the first year after transfer between those who attended the clinic and those who did not. Secondary outcomes were acute rejection, defined as biopsy-proven cellular or antibody-mediated rejection post-transfer according to the Banff criteria [31], and change in estimated glomerular filtration rate (eGFR) in the first and second years following transfer.

The dataset contained information on 32 patients, 16 who attended transfer clinic and 16 who did not. Continuous variables were summarized using means, and categorical variables were summarized as proportions. Bivariate analysis was performed for each variable comparing the two groups. Categorical variables were analyzed using chi-square or Fisher’s exact test (for variables with less than five expected values per cell). Wilcoxon rank-sum test was used to compare continuous variables between the two groups.

All information was obtained from the Organ Transplant Tracking Record (OTTR™, OTTR Chronic Care Solutions, Omaha, NE, USA), a computerized data management system that has been implemented throughout the Multi-Organ Transplant Program at UHN. The OTTR™ application interfaces with the hospital electronic patient record and off-site facilities to provide accessibility to patient data, results, and reports. Pediatric data was obtained from the Hospital for Sick Children electronic patient chart and nephrology clinic database.

## Results

Patient characteristics are summarized in Table 1. All patients were 18 years old at the time of transfer. All had received either a living or deceased donor kidney transplant at the Hospital for Sick Children. There were no significant differences between the groups at baseline (Table 1). In the first year post-transfer, 18.8 % patients who attended the transfer clinic were non-adherent. This compares to 62.5 % of patients who did not attend the transfer clinic. This difference was statistically significant, *p* = 0.03 (Fisher’s exact test).

**Table 1:** Baseline characteristics of the study cohort

Baseline characteristic	Before transfer clinic (*n* = 16)	Attended transfer clinic (*n* = 16)	*p* value
Male (%)	62.5	68.8	NS
Mean age of ESRD diagnosis (years ± SD)	9.7 ± 5.4	13.3 ± 2.8	0.06
Mean age at transplant (years ± SD)	11.0 ± 5.5	13.8 ± 2.8	NS
Mean no. of transplants (Q_25_—Q_75_)	1.0 (1 to 1)	1.0 (1 to 1)	NS
Deceased donor (%)	56.3	62.5	NS
Non-adherence prior to transfer (%)	37.5	50	NS
Serum creatinine at the time of transfer, μmol/l (mean ± SD)	94.8 ± 30.8	105.1 ± 53.8	0.86
eGFR at the time of transfer, ml/min/1.73 m^2^ (mean ± SD)	94.3 ± 29.9	96.4 ± 26.0	0.98

*ESRD* end-stage renal disease, *eGFR* estimated glomerular filtration rate

The number of patients who self-reported medication non-adherence at baseline (defined as self-reported missed medication doses while in pediatric care) was 14/32, 43.8 %. In those who attended the transfer clinic, self-reported medication non-adherence in the first year post-transfer decreased to 2/16 (12.5 %). Self-reported medication non-adherence among patients in the pre-transfer clinic group was 7/16 (43.8 %) in the first year post-transfer, which was unchanged. This too was statistically significant, *p* = 0.049 (Fisher’s exact test).

There was a statistically significant difference in the mean change in eGFR between the two groups in the first year post-transfer, −12.2 ± 14.9 versus −0.9 ± 13.2 ml/min/1.73 m^2^, *p* = 0.045 (Table 2). By 2 years post-transfer, there was no difference in the change in eGFR between groups, −18.4 ± 23.1 versus −13.4 ± 24.6 ml/min/1.73 m^2^, *p* = 0.35.

**Table 2:** Post-transfer outcomes

Outcome	Before transfer clinic	Attended transfer clinic	*p* value
Non-adherent behavior (%)	62.5	18.8	0.03
Self-reported non-adherence (%)	43.8	12.5	0.05
Undetectable CnI levels (%)	18.8	12.5	1.00
Non-attendance at clinic (%)	62.5	25	0.07
Non-attendance for blood tests (%)	56.3	25	0.14
Median change in creatinine in the first year, μmol/l (Q_25_—Q_75_)	11.5 (0 to 16)	−3.5 (−9 to 11)	0.03
Median change in creatinine in the second year, μmol/l (Q_25_—Q_75_)	19.8 (−5 to 12)	11.6 (−4 to 28)	0.63
eGFR 1 year post-transfer, ml/min/1.73 m^2^ (mean ± SD)	93.3 ± 31.9	84.2 ± 25.5	0.98
eGFR 2 years post-transfer, ml/min/1.73 m^2^ (mean ± SD)	81.1 ± 28.7	77.9 ± 24.8	0.81
Change in eGFR 1 year post-transfer, ml/min/1.73 m^2^ (mean ± SD)	−12.2 ± 14.9	−0.9 ± 13.2	0.045
Change in eGFR 2 years post-transfer, ml/min/1.73 m^2^ (mean ± SD)	−18.4 ± 23.1	−13.4 ± 24.6	0.35

*CnI* calcineurin inhibitor, *eGFR* estimated glomerular filtration rate

In the first year after transfer, there was one rejection in each group. The rejection in the patient who did not attend the transfer clinic was associated with documented non-adherence and occurred 8 years post-transplant. This was graded as acute antibody-mediated rejection with C4d positivity, early transplant glomerulopathy, and borderline changes suspicious for acute cellular rejection. One patient in the transfer clinic group experienced a Banff grade 3 acute cellular rejection 3 months post-transplant. This was not associated with non-adherence.

In the second year following transfer, one patient in the transfer clinic group experienced a mixed acute cellular and antibody-mediated rejection with C4d-positivity, transplant glomerulopathy, and a plasma cell-rich infiltrate. This occurred in the context of admitted non-adherence and opioid abuse. There was one graft loss in the transfer group, due to patient death in an accident unrelated to transplant. There were no rejections or graft losses in the group that did not attend the transfer clinic.

## Discussion

This study shows that the addition of an inter-disciplinary transfer clinic, as a one-time intervention, can improve adherence behavior in adolescent kidney transplant recipients during the first year post-transfer to adult care services. Patients who attended the transfer clinic had improved clinic attendance, blood test monitoring, and calcineurin inhibitor levels in the first year post-transfer to adult care. In addition, renal function was better preserved in the first year post-transfer in those who attended the transfer clinic compared to those who did not.

The exact prevalence of non-adherence in kidney transplant patients is difficult to determine, as there is no “gold standard” to assess adherence. In this study, we used a simple composite measure to assess non-adherent behavior. By this definition, 43.8 % of patients were non-adherent at the time of transfer, in keeping with previously reported rates. Patients who attended the transfer clinic were more likely to be adherent with treatment in the year following transfer, with only 3 of the 16 patients (18.8 %) displaying what was considered non-adherent behavior. By contrast, 10 of the 16 (62.5 %) patients who transferred prior to the initiation of the clinic were non-adherent. Self-reported medication non-adherence was also significantly more common among those patients who were transferred before the transfer clinic was in place, 43.8 % compared to 12.5 % in the latter group.

In two studies with a total of 200 kidney transplant patients using electronic monitoring to assess adherence, 20–26 % of patients missed at least 10 % of doses [8, 32]. This correlates well with a meta-analysis of cross-sectional studies using self-report questionnaires, which found a median of 22 % of kidney transplant recipients to be non-adherent [11]. A study using Medicare pharmacy claims in 15,525 kidney transplant recipients allowed the authors to calculate the number of days that a patient did not have medication. This was expressed as a medication possession ratio. Of patients, 25 % had poor adherence based on an overall medication possession ratio of less than or equal to 0.811 over 3 years, analogous to missing 20 % of doses. Patients with adherence in the lowest quartile were 1.8 times more likely to experience graft failure and incurred a $12,840 increase in 3-year medical costs. Adolescent patients age 19–24 were significantly more likely than any other group to be poorly adherent, with an odds ratio of persistent low adherence of 1.56 compared to patients age 24–44 [10].

Prestidge et al. previously demonstrated a reduction in health care costs and improvement in graft outcome over 3 years in 12 patients who received care in a transition clinic compared to a historical cohort [33]. While our primary aim was to examine markers of adherence, we did detect a difference in change in renal function at 1 year; patients who attended the transfer clinic had a significantly lower median increase in creatinine. Patients who were adherent were also more likely to have a lower change in creatinine after 1 year compared to those deemed non-adherent. There was no difference in change in creatinine in the second year post-transplant, and the stability of the effect of the transfer clinic long-term will need to be assessed.

The transfer clinic may have altered adherence for a number of reasons. Patients met with the adult center nephrology staff in the familiar setting of the pediatric hospital, which may have reduced fear and apprehension of transfer. The structure of the clinic also improved communication between the pediatric and adult kidney transplant teams, which may have led to better continuity of care. In addition, the fact that all patients were transferred to a single adult nephrologist and transplant coordinator, as opposed to being distributed among several physicians, may have allowed for the development of some expertise in the adult team when working with this patient population. Nonetheless, compared with other transition interventions, the investment of time and resources in the clinic described here is small [34].

There are a number of important limitations to this study. Our measures of non-adherence may have underestimated its true frequency, but this would be true for both the control and treatment groups. We used non-detectable levels of calcineurin inhibitor as our marker and so may have not detected patients with lower than desired levels due to non-adherence. Non-attendance at clinics and, for blood testing, our other markers are evidence of engagement with treatment rather than a direct measure of medication adherence and may not directly influence graft function. The improvement seen in self-reported non-adherence could be challenged on the basis that as these patients were followed more closely and educated on the importance of medication adherence, they would be less likely to admit to non-adherence. Also, the improvement in adherence behavior may be due to closer follow-up after transfer rather than the transfer process per se. This level of follow-up may not be available outside the setting of a large academic center, which in turn may influence the generalizability of the results. In addition, the improved trajectory of eGFR seen in the first year post-transfer in the intervention group was not maintained after the second year post-transfer. This may be due to the small number of patients in the study or may reflect the fact that the beneficial effects of the transfer clinic wore off over time. Finally, this was not a randomized controlled study, which would be the ideal method with which to test this intervention.

## Conclusions

In summary, kidney transplant recipients who attended an inter-disciplinary transfer clinic were more likely to be adherent with treatment in the year following transfer from a pediatric to an adult care setting than an historical control group. This improvement in adherence was mirrored by better early preservation of renal function. A transfer program should be a priority in any center caring for adolescent kidney transplant recipients. Programs that do not have the luxury of regular adolescent medicine interventions in their clinic may be able to add an important level of preparation with only a single encounter.
